# Cross-pollination affects fruit colour, acidity, firmness and shelf life of self-compatible strawberry

**DOI:** 10.1371/journal.pone.0256964

**Published:** 2021-09-07

**Authors:** Cao Dinh Dung, Helen M. Wallace, Shahla Hosseini Bai, Steven M. Ogbourne, Stephen J. Trueman

**Affiliations:** 1 GeneCology Research Centre, University of the Sunshine Coast, Sippy Downs, Queensland, Australia; 2 School of Science, Technology and Engineering, University of the Sunshine Coast, Sippy Downs, Queensland, Australia; 3 Potato, Vegetable and Flower Research Center – Institute of Agricultural Science for Southern Viet Nam, Thai Phien Village, Da Lat, Lam Dong, Viet Nam; 4 Food Futures Platform, Centre for Planetary Health and Food Security, School of Environment and Science, Griffith University, Nathan, Brisbane, Queensland, Australia; Indian Institute of Science, INDIA

## Abstract

Cross-pollination affects the fruit characteristics of many crops but the effects of cross-pollination on fruit quality of strawberry (*Fragaria × ananassa* Duch.) are poorly known. This study determined how cross-pollination affects fruit quality of the strawberry cultivar, Redlands Joy, under controlled environment conditions. Plants were allocated to one of four treatments, with all flowers on each plant receiving either: (1) unassisted self-pollination (Autogamy); (2) hand-pollination with Redlands Joy pollen (Self); (3) hand-pollination with cross-pollen from a small-fruited cultivar (Sugarbaby); or (4) hand-pollination with cross-pollen from a large-fruited cultivar (Rubygem). Cross-pollination did not significantly affect plant yield or fruit mass, size, shape, firmness or shelf life. However, cross-pollination affected fruit colour and taste attributes. Cross-pollinated fruit were 3%–5% darker than self-pollinated fruit. They also had 26%–34% lower acidity and 43%–58% higher Brix:acid ratio. Cross-pollination by Sugarbaby increased fruit P, K, Ca, Fe and Mn, but decreased B, Cu and Zn, concentrations. Cross-pollination by Rubygem increased fruit Mn, but decreased K and Na, concentrations and reduced shelf life. Fruit mass, length, diameter and firmness within all treatments increased with increasing numbers of fertilized seeds per fruit. Hand self-pollinated fruit had a higher percentage of fertilized seeds than fruit arising from autogamy and they were also darker, redder, firmer, and had a longer shelf life, higher protein concentration, and lower Al and Na concentrations. The results indicate that strawberry fruit quality can be affected by both the source of pollen and the number of stigmas pollinated.

## Introduction

Pollination is vital to sustain food production for the growing human population because more than 75% of food crops require some form of pollination [1]. Furthermore, cross-pollination by a different genotype, compared with self-pollination by the same genotype, has major impacts on global food production because cross-pollination increases yield [2,3] and improves quality attributes such as fruit mass, sweetness, acidity and colour intensity of many horticultural crops [1,4–14]. However, there is limited understanding of how cross-pollination affects yield and quality of some berry crops.

Flowers of strawberry (*Fragaria × ananassa* Duch.) can contain hundreds of free simple carpels, each of which consists of a stigma, a style, and an ovary with a single ovule [15–17]. The carpels are embedded within the receptacle, which enlarges after successful pollination and fertilization of multiple stigmas and ovules, respectively, to form a pseudocarp that is regarded as the strawberry fruit [17,18]. Strawberry flowers can produce fruit with higher mass, sweetness and nutritional value following open-pollination by bees compared with autogamous pollination [19–21]. Strawberry flowers can also produce heavier, redder, firmer, less-malformed and longer-lasting fruit following bee-pollination than autogamous pollination [20,22]. The open- or bee-pollinated fruit possess more fertilized seeds than autogamously-pollinated fruit and this results in heavier fruit and higher yield [20,22,23]. However, it is unclear whether the effects of open- or bee-pollination in these studies were the result of differences in the amount of pollen or in the source of pollen, i.e. cross-pollen *v*. self-pollen, that was deposited on stigmas because the genotypes of the deposited pollen and the resulting seeds were unknown. The effects of different pollen sources on fruit characteristics, including those of the endosperm, embryo, ovary and accessory tissues such as the enlarge receptacle, are termed xenia [24]. There is emerging evidence that cross-pollination can increase strawberry fruit mass [11], but the effects of cross-pollination on other strawberry fruit characteristics remain unknown.

Total soluble solids (TSS), acidity, colour, firmness, shelf life, size and shape are important traits that determine the market acceptance of strawberry fruit [17,25–28]. Strawberry production by the top 20 producing nations alone amounted to over 8 million tonnes of fruit in 2019 [29], and recent attention has focused on maximising the beneficial health components of fruit [30,31]. Strawberry consumption is considered beneficial because strawberry fruit contain essential mineral nutrients [32–34]. A 100-g serving of strawberries can provide 3%–14% of the recommended daily intake of copper, iodine, iron, magnesium, manganese, phosphorus and potassium [35]. Strawberry fruit are also considered beneficial because anthocyanin pigments, associated with the development of red colour during strawberry fruit ripening, function as antioxidants and because strawberry fruit are rich in other beneficial compounds such as vitamin C, phenols and flavonoids [36–39]. The effects of pollination on health components of strawberry fruit also remain unknown.

This study aimed to determine how different pollen sources, including self-pollination and cross-pollination, affect the characteristics of strawberry fruit under controlled conditions. In particular, the study tested the effects of pollination on yield, mass, size, shape, colour, sweetness, acidity, firmness, shelf life and mineral nutrient concentrations of strawberry fruit. Furthermore, it assessed the effects of pollination on the number and percentage of fertilized seeds per fruit and established whether these values affected fruit characteristics. Results from the study will provide greater understanding of the effects of pollen transfer on the quality of strawberry fruit.

## Materials and methods

### Plant material

**Fig 1:**
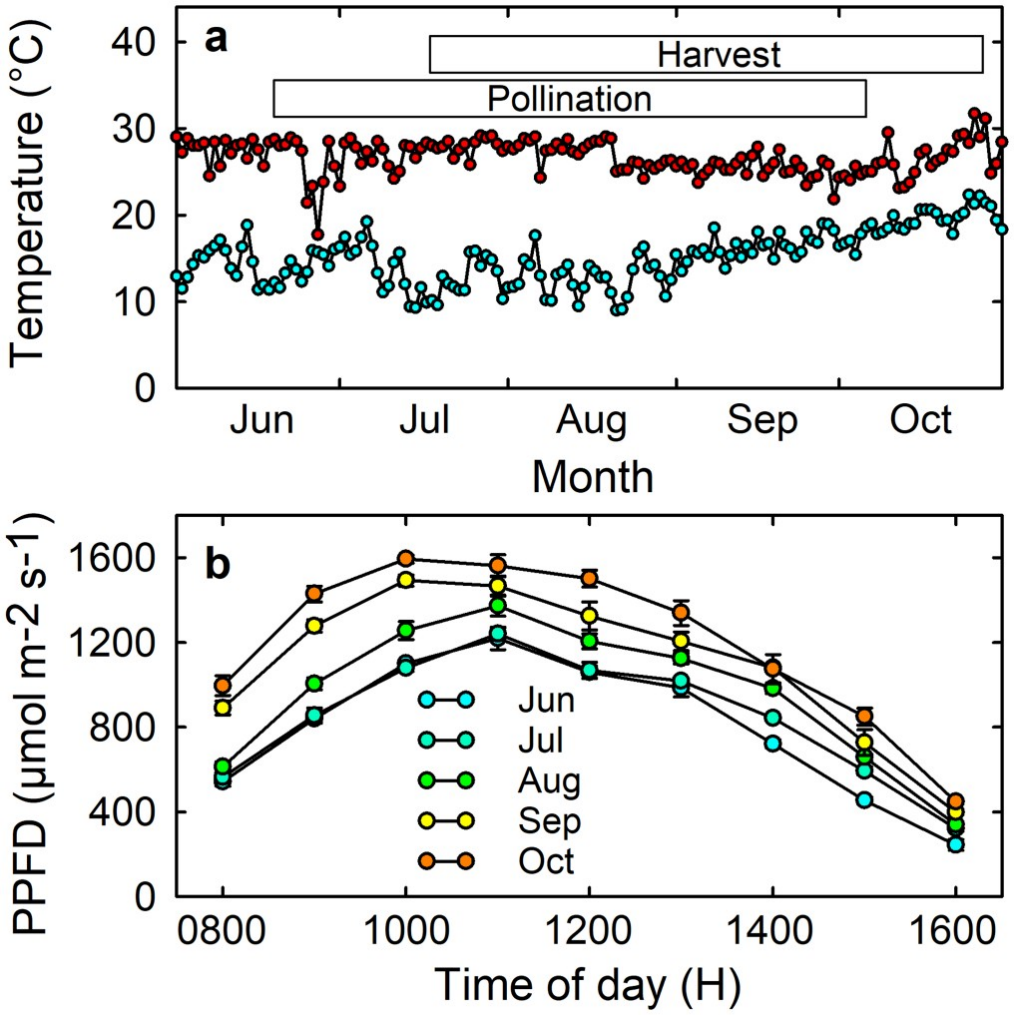
(a) Daily maximum and minimum temperature and (b) mean (± SE) photosynthetic photon flux density (PPFD) in the glasshouse during flowering and fruit set of Redlands Joy, Sugarbaby and Rubygem strawberry. The durations of hand-pollinations of flowers and harvesting of ripe fruit are indicated in panel (a).

Rooted runners of three strawberry cultivars, Redlands Joy, Sugarbaby and Rubygem, were obtained from Sweets Strawberry Runners (Stanthorpe, Australia) in May 2018. These three cultivars arose from a strawberry breeding and selection program in Australia [40–42] that services an industry with approximately 2800 hectares of strawberry plants under cultivation. They have been widely grown commercially and are now available for planting by home gardeners. Fruit of these cultivars are red, medium-glossy, conical to round, of medium firmness, and flavoursome [43–45]. Fruit of Redlands Joy are considered attractive, easily harvested, resistant to rain damage, low-acid, medium-sweetness, and consistently well-liked by consumers [43]. Each runner was transplanted into a 4.5 L pot containing coco-peat (EC < 1 mS/cm, pH 5.5–7.0) and perlite (4:1, v:v) with 2.5 g of Osmocote fertilizer (N:P:K = 19.6:16.0:5.0% w:w, plus trace elements) (Scotts International, Heerlen, The Netherlands). The potted plants were then placed in an enclosed glasshouse at the University of the Sunshine Coast, Sippy Downs, Australia (26°43’S 153°03’E), with plant spacing of 20 cm from pot to pot [46]. Each pot was top-dressed monthly with 15 g of Osmocote fertilizer. A supplementary 5 mL of 1% (v/v) aqueous PowerFeed^®^ foliar fertiliser (Seasol International, Bayswater, Australia) was applied weekly to each plant from transplanting until the commencement of hand-pollination treatments. Irrigation was applied manually to the plant canopy, avoiding direct contact with flowers during the pollination period. Temperatures in the glasshouse (Fig 1a) were recorded using a Tinytag Plus 2 data logger (Gemini Data Loggers, West Sussex, UK). Irradiance was monitored hourly on five cloudless days during the experimental period (Fig 1b) using a quantum sensor (LICOR LI-250A, Lincoln, NE). Pots were rotated frequently to minimise differences in long-term light interception among different sides of each plant. Runners and senesced leaves were removed regularly.

### Experimental design and pollination method

Forty plants of cultivar Redlands Joy with uniform height and crown diameter were arranged in a completely randomized design with four treatments: (1) non-emasculated flowers received unassisted self-pollination (‘Autogamy’); (2) emasculated flowers were hand-pollinated with self-pollen (‘Self’); (3) emasculated flowers were hand-pollinated with Sugarbaby pollen (‘Sugarbaby’); or (4) emasculated flowers were hand-pollinated with Rubygem pollen (‘Rubygem’). Each treatment was applied to all flowers on each of 10 replicate plants from the commencement of flowering in June 2018 until the end of flowering in October 2018.

**Fig 2:**
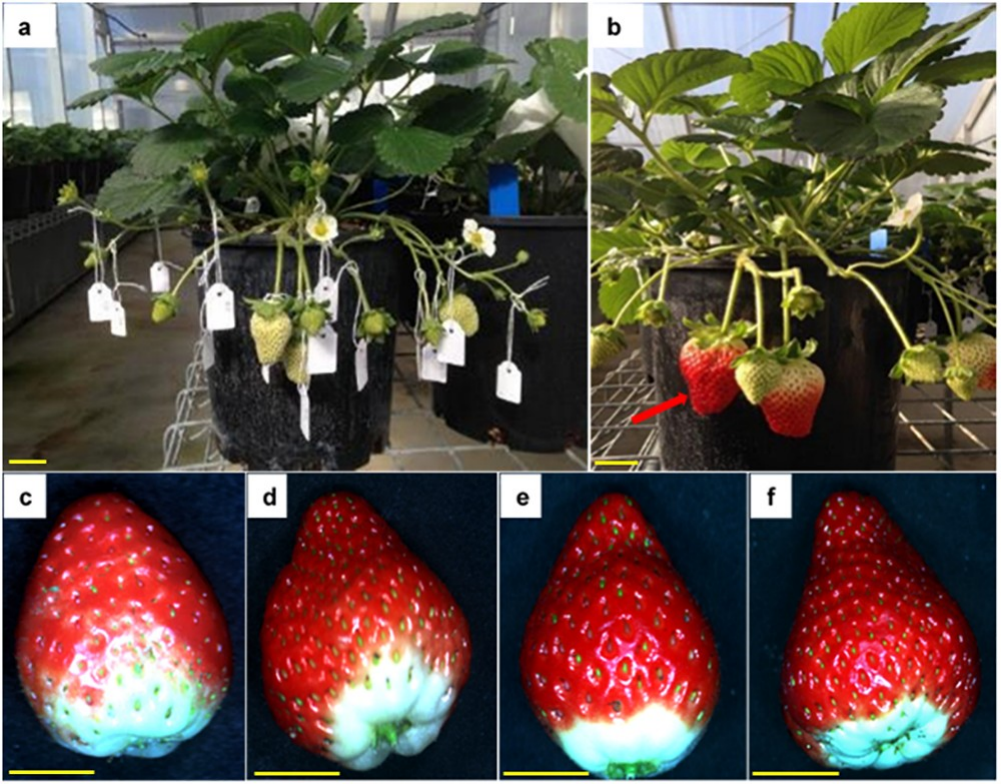
Experimental potted plants of strawberry in the glasshouse, with fruits at (a) immature stage and (b) mature stage (ripe fruit indicated by arrow). Typical colour of cultivar Redlands Joy fruit arising from (c) unassisted self-pollination, or hand-pollination with (d) self-pollen or cross-pollen from (e) cultivar Sugarbaby or (f) cultivar Rubygem. Scale bars = 1 cm. Photographs: Cao Dinh Dung.

Each flower on plants in Treatment 1 (Autogamy) was labelled with a miniature paper tag that recorded the date on which the anthers first released pollen (Fig 2a). The flower was then covered with a fine-mesh bag, which was permeable for water vapour but prevented wind and cross-pollination, from first anther dehiscence until its petals started to fall. Each flower on plants in Treatments 2–4 (‘Self’, ‘Sugarbaby’ or ‘Rubygem’ pollen) had its anthers removed before the flower opened and was then covered with a fine-mesh bag until the stigma was receptive. Mature anthers of each cultivar were harvested daily and dried at glasshouse temperature (Fig 1a) from 1200 H to 1400 H in the lid of a 100-mL plastic jar. Released pollen was then transferred to the plastic jars, which were closed and stored overnight in a refrigerator at 4 °C. Flowers in Treatments 2–4 were hand-pollinated using a paintbrush, with separate brushes used for each pollen-donor cultivar. Each flower in Treatments 2–4 was labelled with a miniature paper tag that recorded the date of first hand-pollination and was covered again with a fine-mesh bag to completely exclude other pollen. Pollination was repeated daily until the petals started to fall.

### Yield, fruit characteristics and shelf life

Each fruit was harvested at commercial maturity; i.e. when the skin was becoming fully red (Fig 2b) and was numbered consecutively. Time to maturity for each fruit was calculated as the number of days from first anther dehiscence (Treatment 1: ‘Autogamy’) or first hand-pollination (Treatments 2–4: ‘Self’, ‘Sugarbaby’ or ‘Rubygem’) to harvest. The fresh mass, length and diameter of each fruit, excluding the pedicel and sepals, were recorded. Length:diameter ratio was then calculated as a measure of shape for each fruit. Yield was calculated as the total fresh mass of all fruit harvested from a plant.

Colour was assessed for each fruit using a CR-10 colorimeter (Konica Minolta, Chiyoda, Japan) in the L*a*b* colour space. L*, a* and b* values indicate brightness (brighter is more positive and darker more negative), redness (redder is more positive and greener more negative), and yellowness (yellower is more positive and bluer more negative), respectively. Firmness of each fruit, with the exception of the twentieth to twenty-fourth fruit harvested from each plant (i.e. fruits 20–24), was determined using a Bareiss Hpe-II fruit penetrometer (Bareiss Prüfgerätebau, Oberdischingen, Germany) with a 3-mm diameter probe. This was the smallest available probe and was recommended by the manufacturer for testing strawberry fruit. Maximum force was recorded when the penetrometer was pushed 6 mm vertically downwards into the fruit apex at a speed of 100 mm min^–1^ [23,47,48]. These fruits were then frozen at -20 °C prior to subsequent quality analyses. The twentieth to twenty-fourth fruit harvested from each plant were stored at 4 °C to determine shelf life. Shelf life was calculated as the number of days until the skin became commercially unattractive due to dark spot or mould development.

Ten frozen fruit from each plant (fruits 1, 6, 11, 16, 19, 26, 31, 36, 41 and 46) were used to measure seed number, TSS concentration and acidity. The total number of seeds (i.e. achenes) and the number of fertilized seeds were counted on each fruit. Unfertilized seeds were visibly distinguishable from fertilized seeds due to their smaller size (Fig 2c–2f). The percentage of seeds that were fertilized was calculated for each fruit. These fruits were then individually defrosted for 60 s (1000 W, 2450 MHz) in a Panasonic NN-CD987W microwave oven (Panasonic, Osaka, Japan) and the released juices were filtered through filter paper. TSS (°Brix) and acid concentration of the filtered extract were determined using a PAL-BX|ACID4 sugar and acidity meter (Atago, Tokyo, Japan) [49–51]. Brix:acid ratio was then calculated for each fruit.

Eight other frozen fruit from each plant (fruits 5, 10, 15, 19, 25, 30, 35 and 40) were used to analyse mineral nutrient concentrations. Nitrogen concentrations of the fruit were determined by combustion analysis using a LECO 928 analyser (LECO, Saint Joseph, MI) [52–54]. Aluminium, boron, calcium, copper, iron, magnesium, manganese, phosphorus, potassium, sodium, sulphur and zinc concentrations were determined by inductively coupled plasma–atomic emission spectroscopy [55].

### Data analysis

Data were analysed using IBM SPSS Statistics v. 26 (IBM, Armonk, NY). Yield data was analysed by 1-way analysis of variance (ANOVA). Fruit quality and shelf life data were analysed using generalized linear models (GLMs). Pollination treatment and plant (nested within pollination treatment) were regarded as main effects, and the date of harvest of each fruit was incorporated as a covariate. Due to highly significant effects of date of harvest, a series of GLMs was used to compare two pollination treatments in each model. Holm-Bonferroni corrections were applied to adjust the significance values for multiple models. Linear regressions were also performed to evaluate effects of the number of fertilized seeds, the percentage of fertilized seeds, the number of unfertilized seeds, or the percentage of unfertilized seeds per fruit as the independent variable and fruit quality parameters as the dependent variable. Treatment differences or regressions were regarded as significant at P < 0.05. Means are presented with standard errors.

## Results

### Pollination effects on fruit yield, fruit quality and seed fertilization

Cross-pollination, when compared with self-pollination, did not significantly affect yield of Redlands Joy strawberry plants (Table 1). Cross-pollination also had no significant effect on individual fruit mass, length, diameter, length:diameter ratio, or time to maturity (Table 1). These parameters also did not differ significantly between self-pollinated fruit and fruit arising from autogamy (Table 1).

**Table 1:** Plant yield and individual size, shape and time to maturity of Redlands Joy strawberry fruit arising from unassisted self-pollination (Autogamy) or hand-pollination with self-pollen (Self) or cross-pollen from another cultivar (Sugarbaby or Rubygem).

Fruit parameter	Pollination treatment			
	Autogamy	Self	Sugarbaby	Rubygem
**Plant yield (g)**	473 ± 24	543 ± 47	507 ± 33	545 ± 26
**Fresh mass (g)**	9.65 ± 0.23	10.07 ± 0.26	9.64 ± 0.24	10.04 ± 0.24
**Length (cm)**	3.03 ± 0.03	3.06 ± 0.03	3.02 ± 0.03	3.04 ± 0.03
**Diameter (cm)**	2.73 ± 0.02	2.75 ± 0.06	2.70 ± 0.03	2.74 ± 0.02
**Length:diameter**	1.11 ± 0.01	1.12 ± 0.01	1.12 ± 0.01	1.11 ± 0.01
**Time to maturity (d)**	27.8 ± 0.2	27.8 ± 0.2	27.8 ± 0.2	27.9 ± 0.2

Means ± SE within a row are not significantly different (1-way ANOVA for yield and GLM for individual fruit parameters; P > 0.05; n = 10 plants for yield and n = 467–532 fruit for other parameters).

**Fig 3:**
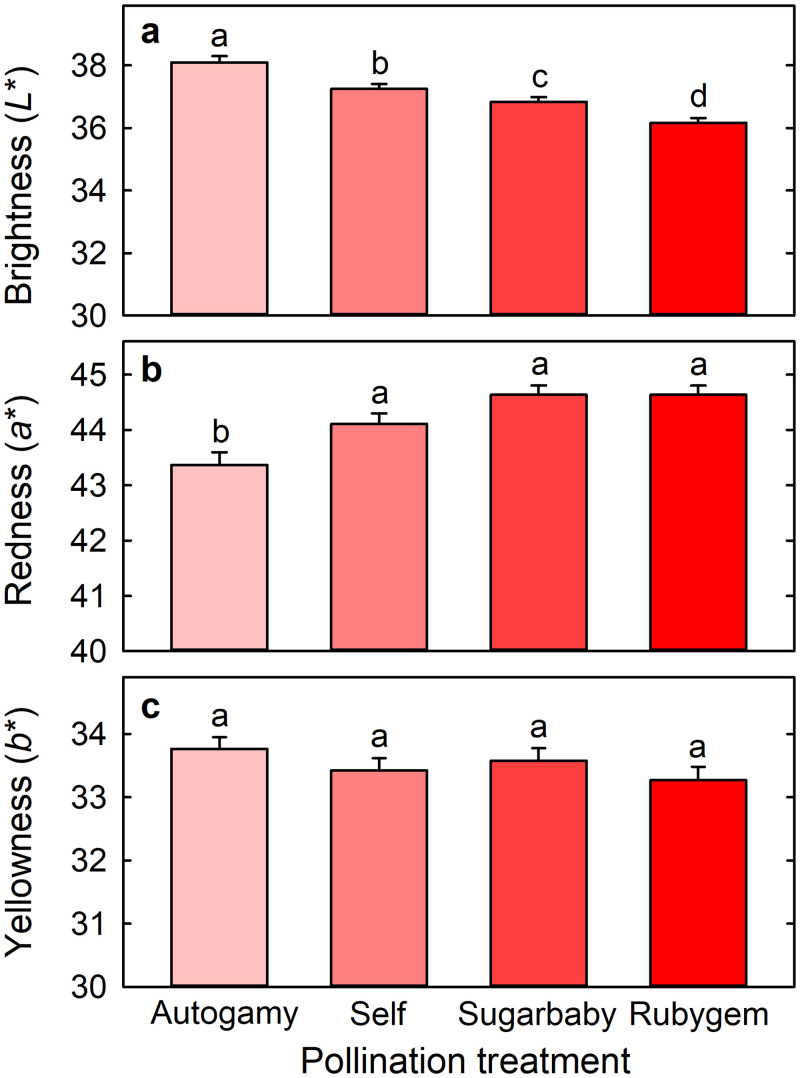
(a) Brightness (L*), (b) redness (a*), and (c) yellowness (b*) of Redlands Joy strawberry fruit arising from unassisted self-pollination (Autogamy) or hand-pollination with self-pollen (Self) or cross-pollen from another cultivar (Sugarbaby or Rubygem). Means + SE with different letters are significantly different (GLM; P < 0.05; n = 460–521 fruit).

Cross-pollinated fruit were darker than self-pollinated fruit, with L* of 36.83 ± 0.15 and 36.17 ± 0.15 following cross-pollination with Sugarbaby or Rubygem, respectively, compared with 37.25 ± 0.16 following self-pollination (Fig 3a). The brightest fruit arose from autogamy, with L* of 38.09 ± 0.20. Fruit redness (a*) and yellowness (b*) did not differ significantly between cross- and self-pollinated fruit (Fig 3b and 3c). However, cross-pollinated fruit (44.63 ± 0.17 and 44.64 ± 0.17) and self-pollinated fruit (44.11 ± 0.19) were all redder than fruit arising from autogamy (43.37 ± 0.23) (Fig 3b).

**Fig 4:**
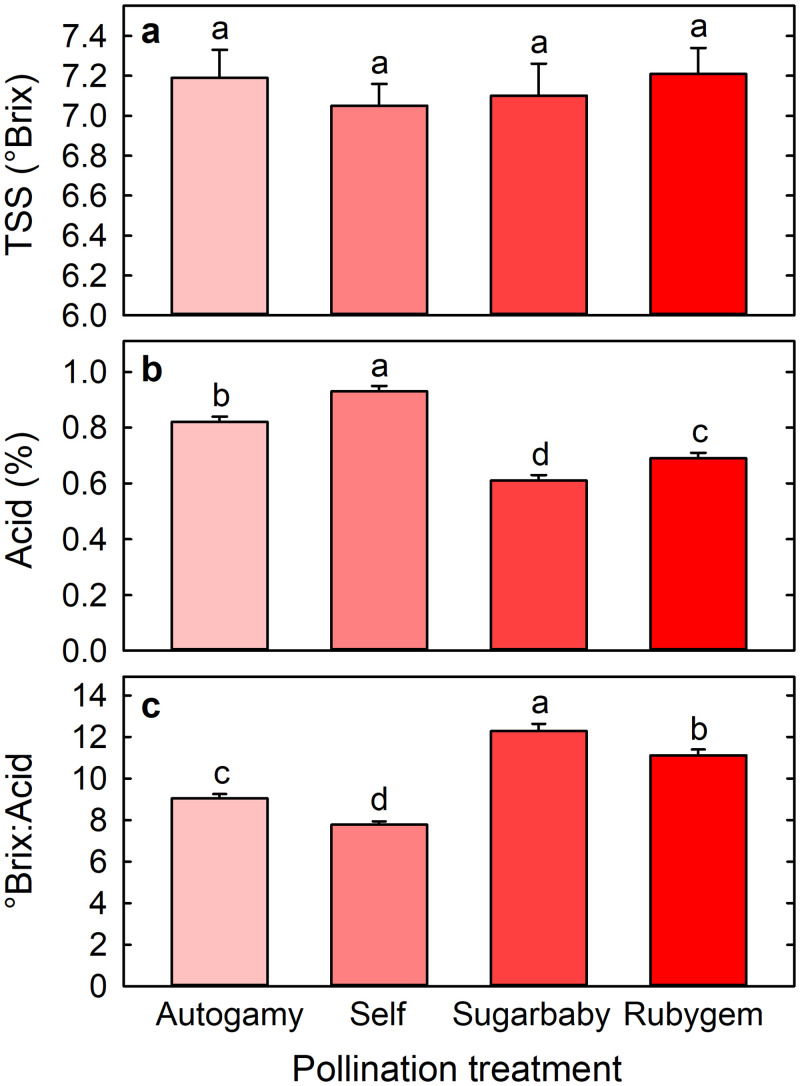
(a) Concentration of total soluble solids (TSS; °Brix), (b) acid (%), and (c) °Brix:acid ratio of Redlands Joy strawberry fruit arising from unassisted self-pollination (Autogamy) or hand-pollination with self-pollen (Self) or cross-pollen from another cultivar (Sugarbaby or Rubygem). Means + SE with different letters are significantly different (GLM; P < 0.05; n = 99–100 fruit).

Cross-pollination greatly reduced fruit acidity without significantly affecting TSS concentration (°Brix) (Fig 4a and 4b). Acid concentrations were 0.61% ± 0.02% and 0.69% ± 0.02% following cross-pollination with Sugarbaby or Rubygem, respectively, compared with 0.93% ± 0.02% following self-pollination. As a result, °Brix:acid ratios were higher following cross-pollination with Sugarbaby (12.3 ± 0.4) or Rubygem (11.1 ± 0.3) than following self-pollination (7.8 ± 0.2) (Fig 4c). Acid concentration (0.82% ± 0.02%) of fruit arising from autogamy was significantly higher than cross- and lower than self-pollinated fruit (Fig 4b). As a result, °Brix:acid ratio (9.0 ± 0.2) was significantly lower than cross- and higher than self-pollinated fruit (Fig 4c).

**Fig 5:**
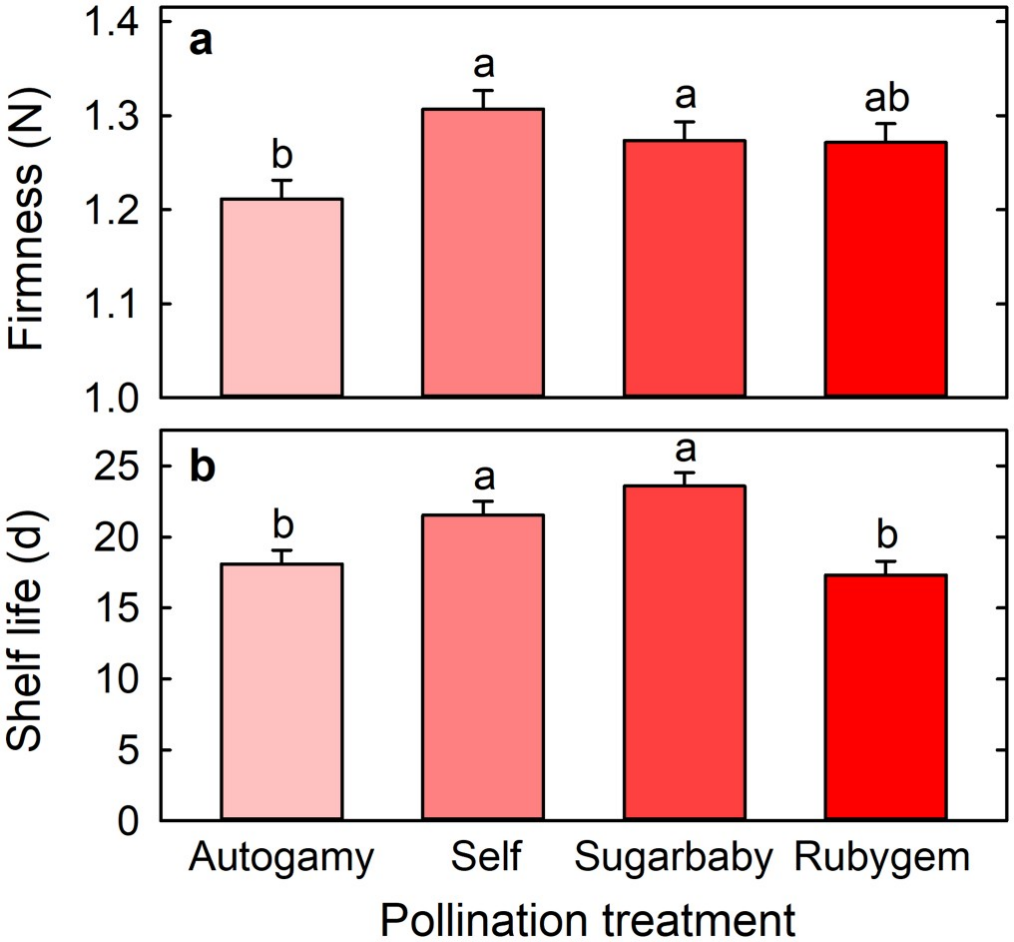
(a) Firmness and (b) shelf life of Redlands Joy strawberry fruit arising from unassisted self-pollination (Autogamy) or hand-pollination with self-pollen (Self) or cross-pollen from another cultivar (Sugarbaby or Rubygem). Means + SE with different letters are significantly different (GLM; P < 0.05; n = 410–467 fruit for firmness and n = 48–50 fruit for shelf life).

Firmness was greater for fruit arising from cross-pollination with Sugarbaby (1.28 N ± 0.02 N) or self-pollination (1.31 N ± 0.02 N) than from autogamy (1.21 N ± 0.02 N) (Fig 5a). Firmness of fruit arising from cross-pollination with Rubygem (1.27 N ± 0.02 N) did not differ significantly from the fruit of any other treatment (Fig 5a). Shelf life was longer for fruit arising from cross-pollination with Sugarbaby (23.6 d ± 0.96 d) or self-pollination (21.5 d ± 0.98 d) than from autogamy (18.1 d ± 0.97 d) or cross-pollination with Rubygem (17.3 d ± 0.96 d) (Fig 5b).

Cross-pollination with Sugarbaby increased fruit Ca, Fe, K, Mn and P concentrations by 32%, 41%, 14%, 16% and 10%, but decreased fruit B, Cu and Zn concentrations by 23%, 31% and 12%, respectively, when compared with self-pollination (Table 2). Cross-pollination with Rubygem increased fruit Mn concentration by 11%, but decreased fruit K and Na concentrations by 5% and 23%, respectively, when compared with self-pollination (Table 2). Fruit arising from autogamy had lower N (by 20%–24%), higher Al (by 30%–45%) and higher Na (by 37%–79%) concentrations than fruit from any hand-pollinated treatment.

**Table 2:** Mineral nutrient concentrations (mg/100g fresh mass) in Redlands Joy strawberry fruit arising from unassisted self-pollination (Autogamy) or hand-pollination with self-pollen (Self) or cross-pollen from another cultivar (Sugarbaby or Rubygem).

Nutrient	Pollination treatment			
	Autogamy	Self	Sugarbaby	Rubygem
**Nitrogen (N)**	170 ± 5b	225 ± 13a	215 ± 6a	217 ± 9a
**Phosphorus (P)**	29.2 ± 0.5a	26.8 ± 0.6b	29.5 ± 0.7a	27.1 ± 0.7b
**Potassium (K)**	189 ± 2b	177 ± 3c	202 ± 3a	168 ± 3d
**Aluminium (Al)**	0.122 ± 0.007a	0.084 ± 0.005b	0.094 ± 0.006b	0.085 ± 0.005b
**Boron (B)**	0.163 ± 0.003a	0.154 ± 0.005ab	0.119 ± 0.004c	0.144 ± 0.003b
**Calcium (Ca)**	55.0 ± 3.0a	38.0 ± 2.3c	50.1 ± 3.1ab	42.4 ± 2.9bc
**Copper (Cu)**	0.029 ± 0.002b	0.035 ± 0.002a	0.024 ± 0.002c	0.031 ± 0.002ab
**Iron (Fe)**	1.41 ± 0.06a	1.08 ± 0.04b	1.52 ± 0.05a	1.11 ± 0.04b
**Magnesium (Mg)**	18.0 ± 0.2a	17.1 ± 0.3bc	17.9 ± 0.3ab	16.6 ± 0.2c
**Manganese (Mn)**	0.591 ± 0.015a	0.476 ± 0.015c	0.552 ± 0.018ab	0.528 ± 0.013b
**Sodium (Na)**	1.81 ± 0.07a	1.32 ± 0.08b	1.31 ± 0.06b	1.01 ± 0.06c
**Sulphur (S)**	13.3 ± 0.6a	11.3 ± 0.6b	12.3 ± 0.8ab	10.1 ± 0.6b
**Zinc (Zn)**	0.185 ± 0.004a	0.173 ± 0.005a	0.152 ± 0.005b	0.173 ± 0.005a

Means ± SE within a row with different letters are significantly different (GLM; P < 0.05; n = 80 fruit).

**Fig 6:**
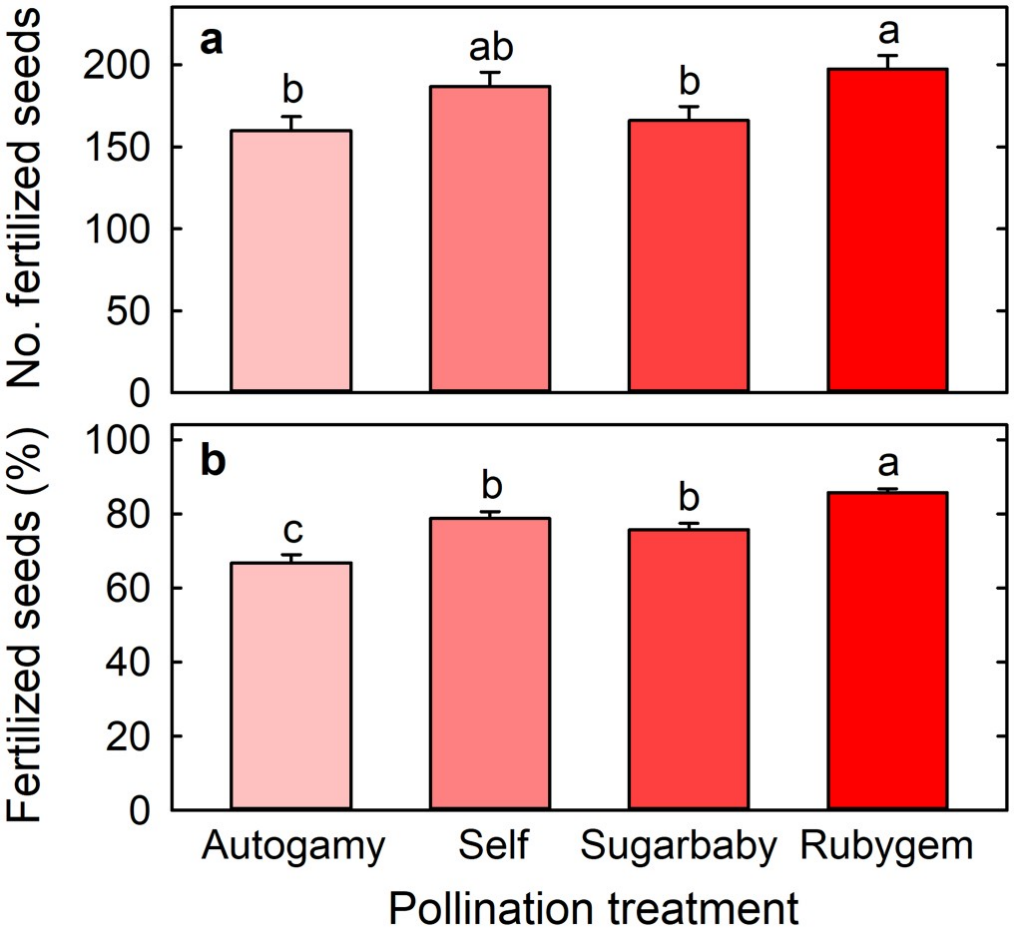
(a) Number of fertilized seeds and (b) percentage of seeds that are fertilized on Redlands Joy strawberry fruit arising from unassisted self-pollination (Autogamy) or hand-pollination with self-pollen (Self) or cross-pollen from another cultivar (Sugarbaby or Rubygem). Means + SE with different letters are significantly different (GLM; P < 0.05; n = 100 fruit).

Cross-pollination, when compared with self-pollination, did not significantly affect the number of fertilized seeds per fruit although fruit arising from Rubygem pollination produced more fertilized seeds (197 ± 8) than fruit arising from Sugarbaby pollination (166 ± 9) (Fig 6a). Fruit arising from cross-pollination with Rubygem also had a higher percentage of fertilized seeds (85.7% ± 1.1%) than fruit arising from cross-pollination with Sugarbaby (75.8% ± 1.7%) or self-pollination (78.8% ± 1.8%) (Fig 6b). Fruit arising from autogamy had a lower percentage of fertilized seeds (66.8% ± 2.2%) than fruit from any other treatment.

### Relationships between fertilized seeds and fruit quality

**Fig 7:**
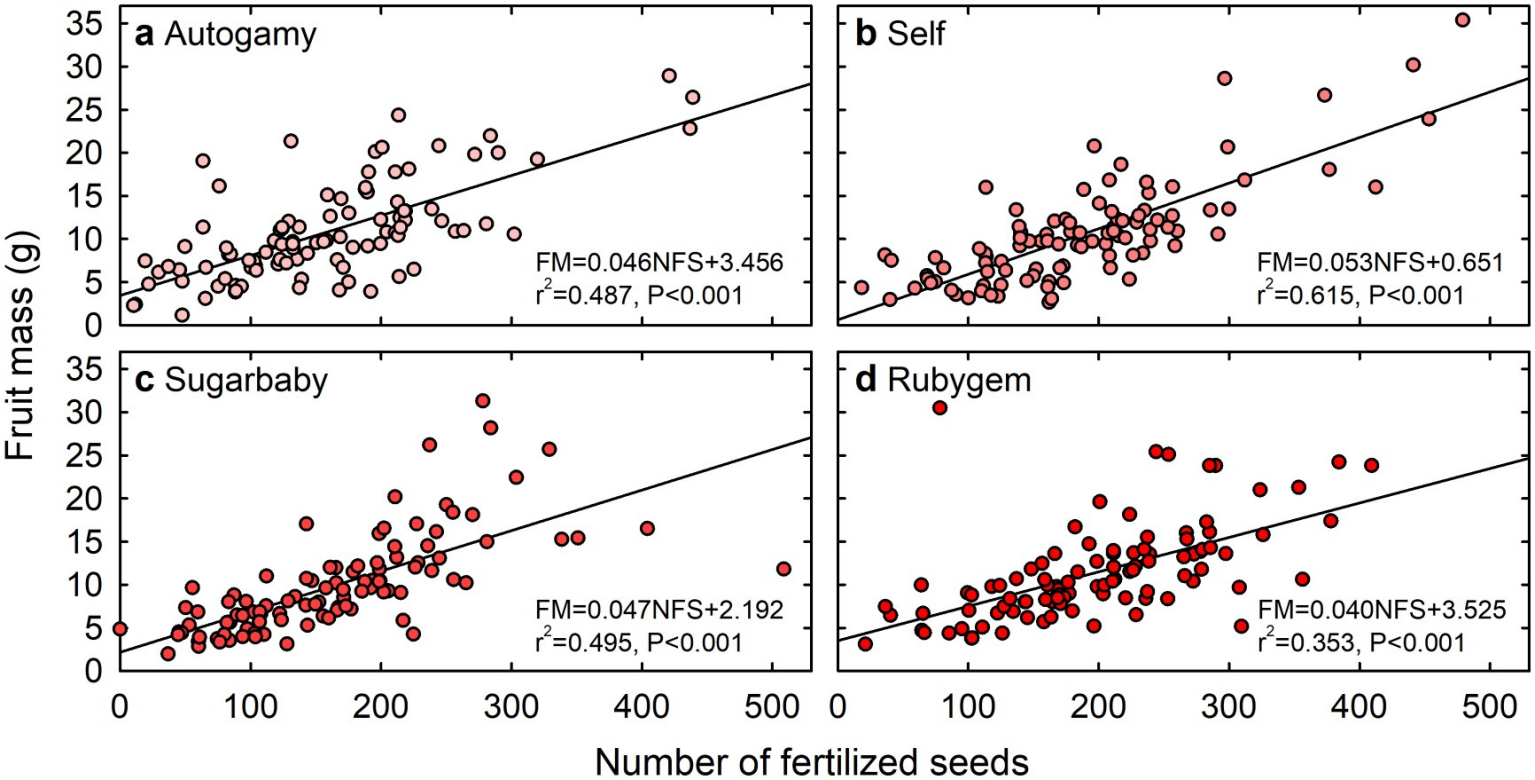
Relationship between the number of fertilized seeds and fresh mass of Redlands Joy strawberry fruit arising from unassisted self-pollination (Autogamy) or hand-pollination with self-pollen (Self) or cross-pollen from another cultivar (Sugarbaby or Rubygem) (linear regression, P < 0.001; n = 100 fruit).

The strongest linear relationships between seed parameters and fruit quality attributes were obtained using the number of fertilized seeds, rather than the number of unfertilized seeds or the percentages of fertilized or unfertilized seeds, as the independent variable. Therefore, only the relationships between number of fertilized seeds and fruit quality attributes are presented (Table 3). Fruit fresh mass (r^2^ values of 0.353–0.615) (Fig 7), length (r^2^ values of 0.064–0.491) and diameter (r^2^ values of 0.113–0.596) were positively and consistently related to the number of fertilized seeds in all treatments (Table 3). Fruit firmness was also related positively to the number of fertilized seeds, although the relationships were not as strong (r^2^ values of 0.043–0.199) (Table 3).

**Table 3:** Coefficients of determination for linear regressions between the number of fertilized seeds and the physical and chemical attributes of Redlands Joy strawberry fruit arising from unassisted self-pollination (Autogamy) or hand-pollination with self-pollen (Self) or cross-pollen from another cultivar (Sugarbaby or Rubygem).

Dependent variable	Treatment							
	Autogamy		Self		Sugarbaby		Rubygem	
	r^2^	P	r^2^	P	r^2^	P	r^2^	P
**Fresh mass (g)**	0.487	***	0.615	***	0.495	***	0.353	***
**Length (cm)**	0.353	***	0.491	***	0.309	***	0.064	*
**Diameter (cm)**	0.299	***	0.596	***	0.274	***	0.113	***
**Length:diameter**	0.048	*	0.013		0.020		0.002	
**Time to maturity (d)**	0.051	*	0.081	**	0.001		0.067	**
Brightness (L*)	0.107	***	0.032		0.053	*	0.065	*
Redness (a*)	0.011		0.027		0.000		0.038	
Yellowness (b*)	0.054	*	0.000		0.064	*	0.005	
**Firmness (N)**	0.066	***	0.199	***	0.043	*	0.056	*
**TSS (°Brix)**	0.025		0.004		0.008		0.005	
**Acid (%)**	0.010		0.003		0.027		0.026	
**°Brix:acid**	0.003		0.010		0.041	*	0.065	*
**Nitrogen (N)**	0.010		0.038		0.001		0.002	
**Potassium (K)**	0.000		0.008		0.001		0.021	
**Phosphorus (P)**	0.005		0.004		0.009		0.014	
**Aluminium (Al)**	0.012		0.025		0.002		0.043	
**Boron (B)**	0.014		0.002		0.002		0.010	
**Calcium (Ca)**	0.001		0.018		0.003		0.063	*
**Copper (Cu)**	0.002		0.002		0.043		0.006	
**Iron (Fe)**	0.004		0.001		0.006		0.003	
**Magnesium (Mg)**	0.001		0.000		0.006		0.008	
**Manganese (Mn)**	0.000		0.003		0.003		0.002	
**Sodium (Na)**	0.004		0.107	**	0.048		0.002	
**Sulphur (S)**	0.060	*	0.000		0.016		0.005	
**Zinc (Zn)**	0.001		0.001		0.001		0.004	

Significant linear regressions are indicated by asterisks (* P < 0.05; ** P < 0.01; *** P < 0.001; n = 80–100 fruit). Nutrient concentrations are expressed in mg/100g fresh mass. Note that all r values were positive.

## Discussion

This study has demonstrated that pollination can affect many of the characteristics of strawberry fruit. In particular, cross-pollination affected fruit colour and taste attributes. Cross-pollinated fruit could be more appealing to traders and consumers because they were darker, sweeter and had higher concentrations of some mineral nutrients. Autogamously-pollinated fruit had a lower percentage of fertilized seeds and were brighter, less red, less firm and had shorter shelf life than fruit arising from hand self-pollination or from hand pollination by at least one of the cross-pollen sources. The results highlight that both pollen source and pollen quantity can influence strawberry fruit quality. However, pollen source and pollen quantity tended to affect different aspects of fruit quality.

Cross-pollination of strawberry flowers did not significantly affect plant yield, fruit mass, fruit size, or the time taken to reach fruit maturity. These results demonstrate that the study cultivar, Redlands Joy, is self-compatible. However, the study results were among the first to show that the pollen source can affect the appearance and flavour attributes of strawberry fruit. Fruit that arose from hand cross-pollination were darker and less acidic than fruit that arose from hand self-pollination or autogamy, but they had similar TSS concentrations and so were sweeter-tasting. Cross-pollination also increases the sweetness of mandarin (*Citrus reticulata* Blanco) and sweet cherry (*Prunus avium* L.) fruit [4,56] and cross-pollination by cultivars that have sweeter-tasting fruit increases the sweetness of cherimoya (*Annona cherimola* Mill.) fruit [57]. Consumers prefer sweet strawberry fruit [25], and so growers might increase the consumer appeal of their crop by planting multiple varieties in close proximity to each other and managing bee populations to maximise cross-pollen transfer. Honey bees tend to forage along rows rather than across rows on strawberry farms and so cross-pollination could be improved further by planting more than one cultivar within the same row [58]. Cultivar Redlands Joy was found to be self-compatible but it remains possible that other cultivars such as Sugarbaby and Rubygem have some degree of self-incompatibility. If that were the case, then enhanced cross-pollination might benefit their yield and fruit size in addition to affecting their appearance and flavour.

Limited pollen deposition also resulted in lower fruit quality. Autogamy significantly decreased the percentage of fertilized seeds per fruit compared with the hand-pollination treatments. This indicates that autogamously-pollinated flowers experienced a pollination deficiency, with insufficient pollen being deposited on the stigmas to fertilize many of the ovules. Pollen deposition was not measured in the current study, but pollination experiments in open fields of strawberry cultivar Albion suggest that hand-pollination provides greater pollen deposition than bee-pollination, and that bee-pollination provides greater pollen deposition than autogamy [58]. Autogamous fruit of Redlands Joy were brighter, less red, less firm and had shorter shelf life than fruit arising from hand self-pollination or cross-pollination by Sugarbaby. Exclusion of insect pollinators from strawberry flowers has resulted previously in fruit with fewer fertilized seeds, higher proportions of fruit deformation, and shorter shelf life [20,21]. Together, these results suggest that autogamous pollination is associated with limited pollen deposition and that this can result in fruit with poor appearance and reduced shelf life, even in self-compatible cultivars like Redlands Joy. Red colour development in strawberry fruit is associated with the accumulation of anthocyanin pigments, which are beneficial for human health [59–63]. Increasing fruit firmness and shelf life play an important role in reducing postharvest losses of strawberry [64].

The current results are the first to show that pollination can change the mineral nutrient concentrations of strawberry fruit. Cross-pollination of Redlands Joy flowers by Sugarbaby increased fruit Ca, Fe, K, Mn and P concentrations while cross-pollination by Rubygem increased fruit Mn concentration. Cross-pollination by Sugarbaby decreased fruit B, Cu and Zn concentrations while cross-pollination by Rubygem decreased fruit K and Zn concentrations. In addition, hand-pollination with either self- or cross-pollen increased N concentrations, and decreased Al and Na concentrations, when compared with autogamous pollination. Nitrogen concentrations are correlated with crude protein concentrations in fruit, with protein concentration being approximately 6 × N concentration [65]. The 26%–32% higher N concentrations in well-pollinated fruit could help consumers to reach the recommended daily protein intake of 20–25 g [66]. However, strawberry fruit do not provide high amounts of dietary protein, with mean protein concentrations in the current study being less than about 1.4%; i.e. less than about 0.14 g per fruit. Interestingly, hand-pollinated strawberry fruit had 27%–56% lower Na concentrations than autogamously pollinated fruit, demonstrating that consumption of well-pollinated fruit could help to reduce salt intake. These results reinforce that cross-pollination affects many fruit characteristics, but also highlight that both the type and the amount of pollen deposited on the stigmas can influence fruit quality.

Fruit mass, length, diameter and firmness were related consistently within all treatments to the number of fertilized seeds per fruit, although the relationship between firmness and number of fertilized seeds was weak. These aspects of fruit quality were not influenced significantly by the source of pollen. Limited pollen deposition can reduce the set of fertilized seeds, and this resulted in smaller and softer fruit. Strawberry fruit growth is regulated by the concentration of indole-3-acetic acid, which is produced by fertilized seeds on developing fruit [23]. Filled seeds may also provide structural support to the skin of the fruit [17]. The number of fertilized seeds can, therefore, affect the phenotype of the fruit. Low seed numbers have been related previously to reduced fruit mass or size, and higher frequencies of fruit deformation, in other strawberry cultivars [11,19–21,23].

Some components of fruit quality that were affected by pollen source, such as acidity, Brix:acid ratio, and manganese and zinc concentrations, were generally not related to the number of fertilized seeds per fruit. Putative paternity effects on fruit characteristics in multi-seeded fruit are sometimes difficult to distinguish from the potentially-confounding effects of the number of fertilized ovules and developing seeds [67]. However, the current results suggest that some cross-pollination effects on the flavour attributes and mineral nutrient concentrations of strawberry fruit were not attributable to the number of fertilized seeds but may have been regulated largely by the paternity of the seeds. These effects on fruit flavour attributes and mineral nutrient concentrations could, therefore, be true xenia effects [24].

## Conclusion

Cross-pollination affected the appearance, flavour attributes and nutrient concentrations of fruit in a self-compatible strawberry variety. Increasing the percentage of fertilized seeds with either hand self- or cross-pollination, compared with autogamy, also increased fruit darkness, redness, protein concentrations, and, for two of the three pollen sources, firmness and shelf life. Improvements in the appearance, taste and dietary composition of fruit can affect marketability because traders and consumers are interested increasingly in healthy, high-quality food. Fresh mass and size of self-compatible strawberry fruit appeared to be determined largely by the number of fertilized seeds whereas other quality attributes such as flavour and some mineral nutrient concentrations appeared to be regulated by seed paternity, independently of the number of fertilized seeds. Strawberry growers need to consider interplanting different cultivars and managing insect pollinators such as bees on farms to ensure the best possible fruit quality.
